# Unforeseeable presentation of *Chryseobacterium indologenes* infection in a paediatric patient

**DOI:** 10.1186/s13104-016-2022-6

**Published:** 2016-04-12

**Authors:** Geethalakshmi Srinivasan, Swapna Muthusamy, Vinod Raveendran, Noyal Mariya Joseph, Joshy Maducolil Easow

**Affiliations:** Department of Microbiology, Sri Venkateshwaraa Medical College Hospital and Research Centre, Ariyur, Puducherry, 605 102 India; Department of Microbiology, JIPMER, Puducherry, 605 005 India

**Keywords:** Case report, Cellulitis, *Chryseobacterium indologenes*, Soft-tissue infection

## Abstract

**Background:**

We report for the first time a case of community acquired *Chryseobacterium indologenes* soft tissue infection in an immunocompetent patient.

**Case presentation:**

A 11 year female child, from South-Asia of Indian origin presented with fever, pain and swelling in right leg for 3 days with no significant past history. Incision and drainage was done and pus was sent for culture and sensitivity. Radiological investigation showed subtle irregular soft tissue density. Pus culture grew multidrug resistant *C. indologenes.*

**Conclusion:**

Though of low pathogenicity, our case emphasises its unpredictable nature and the need to determine minimum inhibitory concentration breakpoints for therapy.

## Background

Previously known as *Flavobacterium*, the genus *Chryseobacterium* comprising six species are non-glucose fermenting bacilli. *Chryseobacterium indologenes* species was first isolated in 1993 from tracheal aspirate of a patient complicated with ventilator associated pneumonia. The clinical significance of the infections due to *C. indologenes* is poorly studied as not many cases are reported in literature and is also a rare human pathogen. Commonly seen in the environment, these are known to cause a spectrum of infections usually in hospitalized and immunocompromised patients, and in infants. Here we describe a case of *C. indologenes* infection in an immunocompetent child.

## Case presentation

**Fig. 1:**
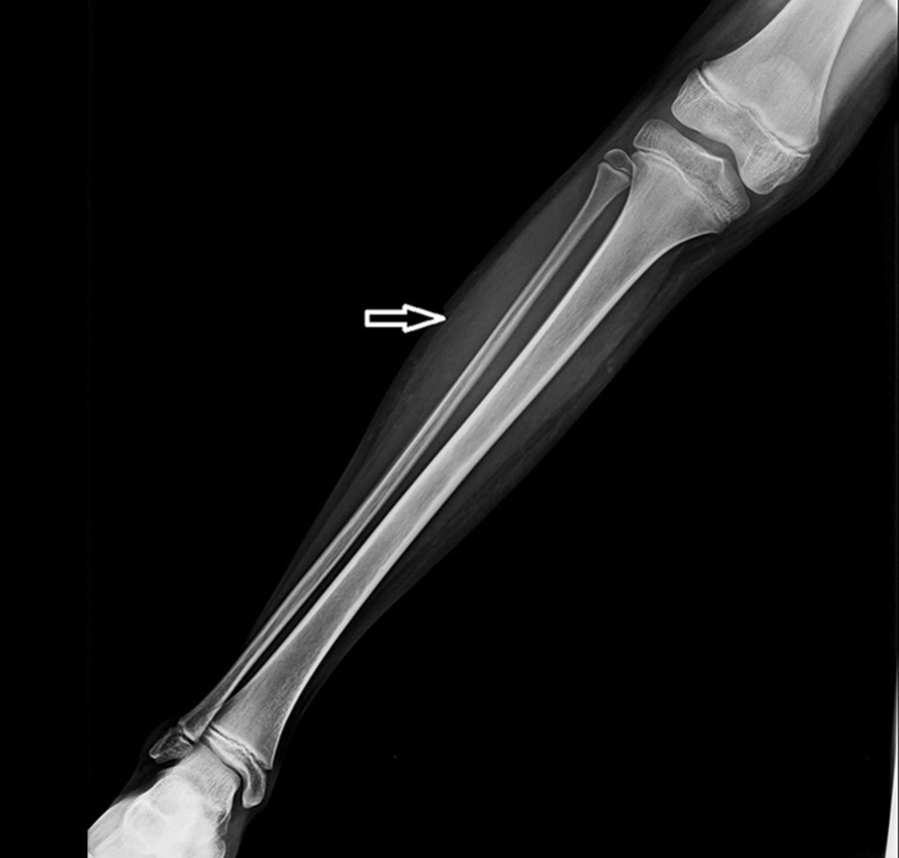
Plain radiograph anteroposterior projection demonstrating patchy irregular increased soft tissue densities in the lateral aspect of the middle third of the right leg as marked by the *arrow*

A 11 year old female child from South-Asia of Indian origin, presented to the orthopaedics department with complaints of fever, non-traumatic pain and swelling in the right leg for about 3 days. There was no significant past history. She was septic and was noted to have a diffuse swelling over the upper third of right leg with skin erythema, warmth and tenderness. No discharging sinuses were noted. Blood investigations showed a raised white cell count and marginally increased erythrocyte sedimentation rate (ESR). Rest of the haematological and biochemical markers were within normal limits. Blood sample was sent for culture and sensitivity which showed no growth. Anteroposterior and lateral views of plain radiograph of the right leg showed normal tibia and fibula with subtle irregular altered soft tissue density in the lateral aspect of the mid third of the leg (Fig. 1). Incision and drainage was performed the following day under general anaesthesia. Thick purulent discharge was drained and about 5 ml of the discharge was sent for culture and sensitivity. The wound was left open and dressed appropriately (Table 1).

**Table 1:** Timeline of case management

Time	Process followed	Observation and management
Day 1	History	Fever, pain and swelling for 3 days
	Physical examination	Swelling with erythema, warmth and tenderness
	Blood count, erythrocyte sedimentation rate	Raised white cell count Marginally elevated erythrocyte sedimentation rate
	X-ray	Normal tibia and fibula with subtle irregular altered soft tissue density in the lateral aspect of the mid-third of the leg
	Blood culture	No growth after 7 days of incubation
	Treatment	Patient was empirically given parenteral cefotaxime, metronidazole and gentamicin
Day 2	Incision and drainage under general anaesthesia	Thick purulent discharge was obtained and sent for culture and sensitivity
	Gram stain	Plenty of pus cells and gram negative bacilli were seen
	Culture	On incubation
Day 3	Genus identification by conventional method	*Chryseobacterium* spp. Subjected for VITEK-2 identification and antibiotic sensitivity testing
Day 4	Species identification by conventional and VITEK-2	*C. indologenes* Minimum inhibitory concentration values by VITEK-2 as in Table 2
Day 5	Switching of antibiotics	Cefotaxime was changed over to ceftazidime Gentamicin was discontinued
Day 5–12	Local examination	Wound was healing well, secondary closure done and patient was discharged

**Fig. 2:**
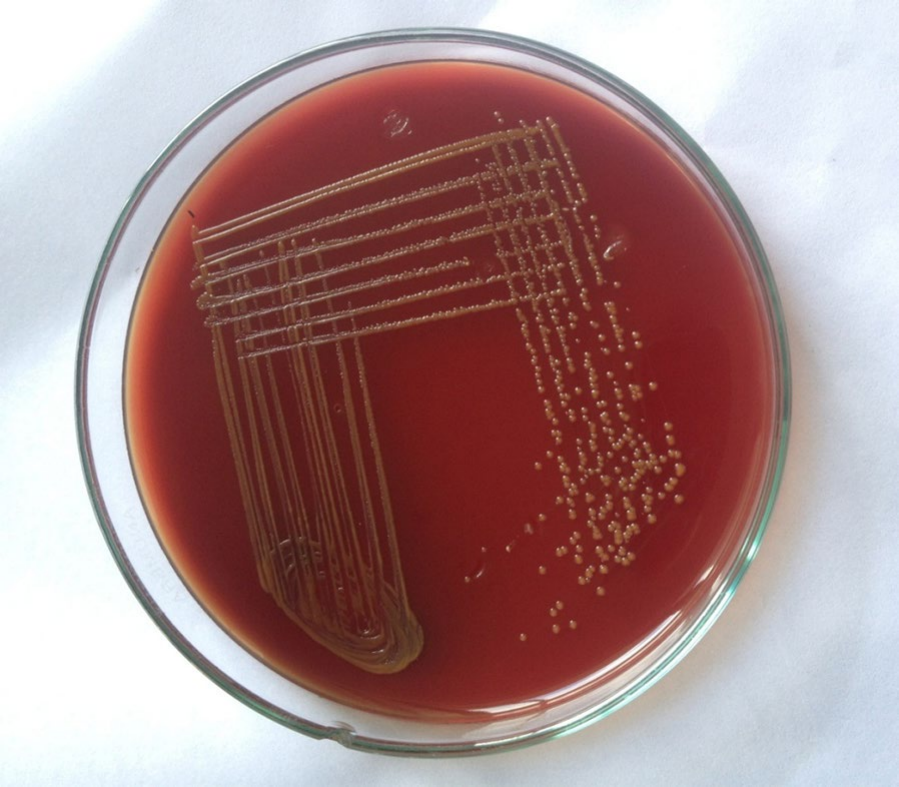
Blood agar showing non-haemolytic *yellow* pigmented colonies of *C. indologenes*

**Fig. 3:**
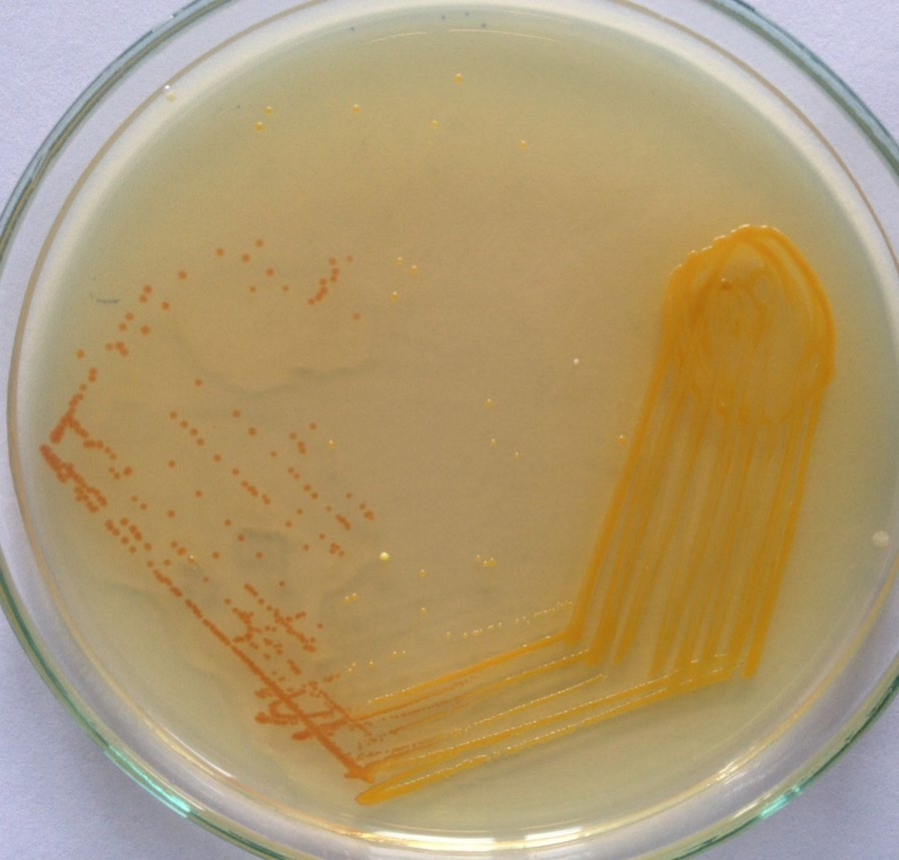
*Yellow* pigmented colonies of *C. indologenes* turning to *red* upon addition of 10 % potassium hydroxide

Gram staining of the purulent material showed numerous pus cells and gram negative bacilli. Inoculation in blood agar showed dark yellow pigmented colonies with no hemolysis (Fig. 2). On nutrient agar yellow pigmented colonies were seen and were noticed to turn to red when 10 % potassium hydroxide (KOH) was added on to the culture (Fig. 3). It did not grow in Mac Conkey agar. The isolate was non-motile, catalase and oxidase positive, indole positive, methyl red negative and Voges–Proskauer’s negative. Urea was not hydrolysed and citrate was not utilized. On triple sugar iron agar, glucose was utilised with gas production. The organism was identified by both conventional method and VITEK-2 systems version: 07.01 as *C. indologenes*.

Genus was presumptively identified as Chryseobacterium after 24 h and genus confirmation, species identification and sensitivity results were available in 48 h. The minimum inhibitory concentration (MIC) values by VITEK-2 system are in Table 2.

**Table 2:** Minimum inhibitory concentration values by VITEK-2 system

Antibiotic	Minimum inhibitory concentration value (µg/ml)	Interpretation
Gentamicin	≥16	Resistant
Amikacin	≥64	Resistant
Ceftazidime	4	Susceptible
Piperacillin–tazobactam	64	Intermediate
Cefaperazone–sulbactam	32	Intermediate
Imipenem	1	Susceptible
Meropenem	≥16	Resistant
Doripenem	≥8	Resistant
Minocycline	≤1	Susceptible
Levofloxacin	0.25	Susceptible
Ciprofloxacin	≤0.25	Susceptible
Cotrimoxazole	≤20	Susceptible
Aztreonam	≥64	Resistant

Our isolate was susceptible to imipenem and resistant to meropenem and doripenem. *Chryseobacterium* species are intrinsically resistant to most beta lactams including carbapenems due to the production of chromosomally mediated metallobetalactamases (MBL). The isolate was a MBL producer which was confirmed by double disk diffusion method with ethylene diamine tetraacetic acid (EDTA).

Patient was empirically started on parenteral cefotaxime, metronidazole and gentamicin, was changed over to intravenous ceftazidime 1 g twice daily and metronidazole infusion 500 mg thrice daily for 5 days based on the sensitivity report following which significant clinical improvement was noted. Subsequently secondary closure was done 10 days later. The wound healed well and the patient was discharged on oral antibiotics with follow up. Consent for publication was obtained from the patient’s parent.

## Discussion

Infection due to *C. indologenes* in humans is a rare occurrence and is usually nosocomial. These are mainly found in soil, plants, water and foodstuffs. They are not found in human flora. Within the hospital premises, they exist in water systems and wet surfaces and can survive in chlorinated water [1].

*Chryseobacterium* species are gram-negative, aerobic, non-motile, oxidase positive, catalase positive, indole positive, producing a distinct yellow orange pigment due to production of flexirubin. Indole was produced in tryptophan broth. The genus *Chryseobacterium* belongs to the family *Flavobacteriaceae*. The commonly isolated species includes *C. meningosepticum*, *C. multivorum*, *C. odoratum*, *C. breve* and group IIb *Chryseobacterium* species which includes *C. indologenes* and *C. gleum*. Of these *C. meningosepticum* is the most pathogenic causing neonatal meningitis [2].

Chryseobacterium infections are frequently associated with the presence of indwelling devices like intra-vascular catheters etc., in immunocompromised patients or in patients on long-term broad-spectrum antibiotics. Factors influencing the development of Chryseobacterium infection include a suitable entry port, invasive procedures, neutropenia, production of biofilm on foreign materials, prolonged use of antibiotics and immunodeficiency [3, 4]. Infections associated with *Chryseobacterium* species include bacteremia, wound sepsis, indwelling device associated infections, ocular infections and intra-abdominal infections. In our patient it was associated with cellulitis, soft tissue infection and abscess formation. They are usually isolated from clinical specimens but rarely from blood as were in our case [4].

Most of the *C. indologenes* infections described in literature were hospital acquired and were seen in patients with underlying debilitating diseases [4–9]. Mostly our isolate is community acquired, as the sample grew Chryseobacterium from pus collected intraoperatively on the second day of admission. During the above said period, environmental surveillance samples did not grow Chryseobacterium.

Interestingly our patient was not immunosuppressed or was on any long term antibiotics or with indwelling devices, making this case unusual in presentation. Douvoyiannis et al. [10] had reported the first case of *C. indologenes* in a 33 day old infant in 2010. Hendaus et al. [11] reported the second case of *C. indologenes* infection from healthy newborn in 2013. Ours would be the third case of *C. indologenes* infection reported from an immunocompetent paediatric patient.

Not much data is available in literature on the choice of appropriate antibiotic for empirical treatment in *C. indologenes* infections [12]. This uncertainty is attributed to the wide spectrum of antimicrobial resistance, lack of gold standard susceptibility testing, unpredictable nature and non-establishment of MIC breakpoints for these organisms [13]. Further, biofilm and proteases production by *C. indologenes* species reduces its antimicrobial susceptibility [14]. Based on the results of SENTRY antimicrobial surveillance program during 1997–2001, the highest prevalence of Chryseobacterium was seen among the elderly. The most appropriate antibiotics that can be used for treating Chryseobacterium infections are newer fluoroquinolones (MIC 90–1 µg/ml) followed by rifampin (MIC 90–2 µg/ml). *C. indologenes* isolates showed adequate susceptibilities to trimethoprim-sulfamethoxazole, ciprofloxacin and piperacillin–tazobactam [15]. Variable susceptibility to vancomycin has been reported in literature [16].

*Chryseobacterium indologenes* is invariably resistant to aminoglycosides, shows varying degree of resistance to carbapenems, cephalosporins, piperacillin–tazobactam and are susceptible to fluroquinolones and cotrimoxazole. Based on the sensitivity pattern reported in SENTRY [15] and other literatures [9, 10, 17], there was no evident difference for community acquired isolates as compared with hospital acquired strains.

In this study, the isolate was resistant to meropenem and susceptible to imipenem by VITEK-2 systems. This could be due to different mechanisms involved in imipenem and meropenem resistance. Meropenem resistance is due to efflux systems and imipenem resistance is by porin mutations. Over expression of MexAB-OprM, an efflux system pumps out meropenem but not imipenem [18]. As the isolate was confirmed to be a MBL producer by imipenem-EDTA double disc diffusion test, all the carbapenems were reported as resistant for therapeutic purpose.

Disk diffusion methods are not reliable and thus susceptibility testing by broth micro dilution method should be performed [19].

## Conclusion

Listed as one of the nosocominal infections, *C. indologenes* infections known for its rare and sporadic incidence can cause potentially serious infections in humans. As described they can cause significant infections in immunocompetent individuals as well. More extensive and long term studies are required to understand the demographics, pathogenicity, virulence factors, arbitrary antimicrobial resistance and appropriate antimicrobial therapy.

## Abbreviations

ESR: erythrocyte sedimentation rate
EDTA: ethylene diamine tetraacetic acid
MBL: metallo beta-lactamase
MIC: minimum inhibitory concentration
KOH: potassium hydroxide

## References

[1] Calderón G, García E, Rojas P, García E, Rosso M, Losada A (2011). *Chryseobacterium indologenes* infection in a newborn: a case report. J Med Case Rep.

[2] Lin YT, Jeng YY, Lin ML, Yu KW, Wang FD, Liu CY (2010). Clinical and microbiological characteristics of *Chryseobacterium indologenes* bacteremia. J Microbiol Immunol Infect.

[3] Christakis GB, Perlorentzou SP, Chalkiopoulou I, Athanasiou A, Legakis NJ (2005). *Chryseobacterium indologenes* non-catheter-related bacteremia in a patient with a solid tumor. J Clin Microbiol.

[4] Hsueh PR, Hsiue TR, Wu JJ, Teng LJ, Ho SW, Hsieh WC (1996). *Flavobacterium indologenes* bacteremia: clinical and microbiological characteristics. Clin Infect Dis.

[5] Akay M, Gunduz E, Gulbas Z (2006). Catheter-related bacteremia due to *Chryseobacterium indologenes* in a bone marrow transplant recipient. Bone Marrow Transplant.

[6] Cascio A, Stassi G, Costa GB, Crisafulli G, Rulli I, Ruggeri C (2005). *Chryseobacterium indologenes* bacteraemia in a diabetic child. J Med Microbiol.

[7] Hsueh PR, Teng LJ, Yang PC, Ho SW, Hsieh WC, Luh KT (1997). Increasing incidence of nosocomial *Chryseobacterium indologenes* infections in Taiwan. Eur J Clin Microbiol Infect Dis.

[8] Hsueh PR, Teng LJ, Ho SW, Hsieh WC, Luh KT (1996). Clinical and microbiological characteristics of *Flavobacterium indologenes* infections associated with indwelling devices. J Clin Microbiol.

[9] Aydin Teke T, Oz FN, Metin O, Bayhan GI, Gayretli Aydin ZG, Oguz M (2014). *Chryseobacterium indologenes* septicaemia in an infant. Case Rep Infect Dis.

[10] Douvoyiannis M, Kalyoussef S, Philip G, Mayers MM (2010). *Chryseobacterium indologenes* bacteremia in an infant. Int J Infect Dis.

[11] Hendaus MA, Zahraldin K (2013). *Chryseobacterium indologenes* meningitis in a healthy newborn: a case report. Oman Med J.

[12] Green BT, Nolan PE (2001). Cellulitis and bacteraemia due to *Chryseobacterium**indologenes*. J Infect.

[13] Clinical and Laboratory Standards Institute (CLSI). Performance standards for antimicrobial susceptibility testing. In: 17th informational supplement. CLSI Document M100-S17. Wayne, PA: Clinical and Laboratory Standards Institute; 2007.

[14] de Ferreira RS, Brandao FF, Lobo SM (2010). *Chryseobacterium indologenes* infection: a case report. Rev Bras Ter Intensiva.

[15] Kirby JT, Sader HS, Walsh TR, Jones RN (2004). Antimicrobial susceptibility and epidemiology of a worldwide collection of *Chryseobacterium* spp: report from the SENTRY antimicrobial surveillance program (1997–2001). J Clin Microbiol.

[16] Bhuyar G, Jain S, Shah H, Mehta VK (2012). Urinary tract infection by *Chryseobacterium indologenes*. Indian J Med Microbiol.

[17] Cunha V, Ferreira M, Fonseca A, Diogo J (2014). Community-acquired *Chryseobacterium indologenes* in an immunocompetent patient. JMM Case Rep.

[18] Rodriguez-Martinez JM, Poirel L, Nordmann P (2009). Molecular epidemiology and mechanisms of carbapenem resistance in *Pseudomonas aeruginosa*. Antimicrob Agents Chemother.

[19] Fraser SL, Jorgensen JH (1997). Reappraisal of the antimicrobial susceptibilities of *Chryseobacterium* and *Flavobacterium* species and methods for reliable susceptibility testing. Antimicrob Agents Chemother.

